# Pathogenic differences of the entomopathogenic fungus *Isaria cateniannulata* to the spider mite *Tetranychus urticae* (Trombidiformes: Tetranychidae) and its predator *Euseius nicholsi* (Mesostigmata: Phytoseiidae)

**DOI:** 10.1007/s10493-018-0247-x

**Published:** 2018-04-02

**Authors:** Xiao-na Zhang, Jian-jun Guo, Xiao Zou, Dao-chao Jin

**Affiliations:** 1 Institute of Entomology, The Provincial Key Laboratory for Agricultural Pest Management of Mountainous Region, The Provincial Special Key Laboratory for Development and Utilization of Insect Resources, Scientific Observing and Experimental Station of Crop Pest in Guiyang, Ministry of Agriculture, Guizhou University, Guiyang, 550005 Guizhou, China; 2 Research Center of Buckwheat Industry Technology, Guizhou Normal University, Guiyang, 550005 Guizhou China; 3 Institute of Fungal Resources, Guizhou University, Guiyang, 550025 Guizhou China

**Keywords:** *Isaria cateniannulata*, *Euseius nicholsi*, *Tetranychus urticae*, Infection process

## Abstract

*Isaria cateniannulata* and *Euseius nicholsi* are two important biological control agents currently being used in many areas of China to control a variety of pests. In order to determine the possibility of a concomitant application with the two agents in a biocontrol program involving the two-spotted spider mite, *Tetranychus urticae*, we quantified the pathogenicity of a strain of *I. cateniannulata* (08XS-1) against females of both *T. urticae* and *E. nicholsi*. We observed the infection process using scanning electron microscopy and fluorescence microscopy to distinguish differences in fungal performance. The female mites were infected by *I. cateniannulata* at 2 × 10^7^ conidia/ml. The mortality of *T. urticae* was 100% when treated with submerged conidia and 92% when treated with aerial conidia (spray), and that of *E. nicholsi* was 4.2 and 6.7%, correspondingly. Following infection with aerial or submerged conidia, mated *E. nicholsi* females displayed no significant differences between treatments and control, indicating the fungus had no obvious effect on their vitality and fertility. This demonstrates that *I. cateniannulata* is safe to *E. nicholsi* when used to control *T. urticae*. The two types of propagules of *I. cateniannulata* are readily produced by common culture, and the submerged conidia, because of their substantially higher mortality, are preferable to the aerial conidia. Our results indicate that *I. cateniannulata* and *E. nichol*si are viable candidates to be concomitantly applied in the biocontrol programs of *T. urticae*.

## Introduction

The two-spotted spider mite, *Tetranychus urticae* Koch (Trombidiformes: Tetranychidae), is one of the most economically important mite species. It is not only one of the most polyphagous herbivores, feeding on more than 1110 plant species belonging to more than 150 plant families (Migeon and Dorkeld 2015), but also a notorious pest for its ability to rapidly develop resistance to many chemicals that have been widely used for mite control (Ghadamyari et al. 2008; Van Leeuwen et al. 2010; Mahdavi Moghadam et al. 2012).

*Euseius nicholsi* (Ehara et Lee) (= *Amblyseius* [*Amblyseius*] *nicholsi*) (Acari: Phytoseiidae) is an important indigenous predator of several species of pest mites and insects in China, and has been successfully employed for reducing populations of *T. urticae* on various crops, such as strawberry and kidney bean (Chen et al. 1994, 1996; Zheng and Jin 2008; Hu et al. 2007). Its control efficiency on pest mites, however, is somewhat limited because the predatory mite prefers to prey on the egg and larval stages of the mites (Chen et al. 1994; Hu et al. 2007).

*Isaria cateniannulata* (Liang) Samson & Hywel-Jones (= *Paecilomyces cateniannulatus* [Liang]) (Hypocreales: Clavicipitaceae) has been isolated from a variety of insects and showed itself one of the most prevalent entomopathogenic fungi. It is the second most common and widely distributed species after *Beauveria bassiana* (Bals.-Criv.) Vuill. in forest ecosystems (Zhang et al. 2007a, b; Li et al. 2011). In our recent papers, we have discussed the efficacy of *I. cateniannulata* in controlling *T. urticae* while having little, if any, detrimental effects on *E. nicholsi* (Zhang et al. 2013, 2014, 2016).

Numerous efforts have been made to control *T. urticae* biologically to reduce the chemical application and its resistance to the pesticides commonly used. During the past decade, a number of studies had demonstrated that several entomopathogenic fungi, including *B. bassiana*, *Metarhizium anisopliae* (Metchnikoff) Sorokin, *Isaria fumosorosea* Wize and *I. cateniannulata*, could cause high mortalities of *T. urticae* under laboratory conditions (Shi et al. 2008; Shi and Feng 2009; Zhang et al. 2013; Ullah and Lim 2015; Wu et al. 2016a, b). The predatory mite *E. nicholsi* had also shown potential as a biological control agent of *T. urticae* (Zheng and Jin 2008, 2009). We previously demonstrated that *I. cateniannulata* has a high efficiency against *T. urticae* and negligible effects on *E. nicholsi* (Zhang et al. 2014, 2016).

Most previous studies have been designed to evaluate the effects of pathogens on pest mites and their predator mites through either direct or indirect approaches, such as: exposing the pest mites and predators to a pathogen and then documenting their mortality and behavior, feeding the predator fungus-infected prey and then examining their infection status, or assessing predator and prey abundance in experimental crops after application of fungal pathogens (Balazy et al. 2008; Zhang et al. 2014). Although previous studies have demonstrated that predators and pests often differ in their mortality after exposure to entomopathogenic fungi (Wu et al. 2016a, b), the causes for the differences remain unclear.

Little information is currently available on differences in pathogenicity of *I. cateniannulata* to *T. urticae* and *E. nicholsi*, we initiated a comparative study on the infecting processes and micromorphological behavior of submerged conidia versus aerial conidia of *I. cateniannulata* on *T. urticae* and *E. nicholsi.* As in many other fungal entomopathogens, the submerged and aerial conidia in cultures of *I. cateniannulata* are the two propagules regularly used on target pests (Zhang et al. 2016). The primary goal of our investigation was to enhance our understanding of the causes for the differences in pathogenicity of *I. cateniannulata* conidia to *T. urticae* and *E. nicholsi*, and to enrich our knowledge in the complex interactions between the two biological control agents. Ultimately, it is hoped that the data generated will contribute to successful concomitant applications of the two agents in biocontrol programs against *T. urticae*.

## Materials and methods

### Collection and preparation of *Isaria cateniannulata*

The strain, 08XS-1, of *I. cateniannulata* was provided by the Institute of Fungal Resources, Guizhou University, Guiyang, China. It was originally isolated from lepidopterous pupae in Xishan, Kunming, Yunnan, in 2008, and maintained on Potato Dextrose Agar medium (PDA). The aerial conidia were produced on PDA at 22  ± 1 °C under 12L:12D for 2 weeks. The submerged conidia were produced in a liquid medium containing Potato Dextrose (without agar) containing 10 glass beads at 22  ± 1 °C under 12L:12D, followed by 200 r/min for 5 days. Aerial conidia and submerged conidia were then prepared into suspension solutions, and the conidium concentrations were determined using a hemocytometer. The conidial concentrations were adjusted to 2 × 10^7^ conidia/ml by adding sterile water containing Tween-80 at 0.05% (v/v). The viability of the aerial and submerged conidia was confirmed on PDA (or PD, without agar) at > 90% RH, and found to exceed 90% (Zhang et al. 2014, 2016).

### Collection and rearing of mite colonies

*Euseius nicholsi* and the prey *T. urticae* mites were obtained from colonies maintained at the Provincial Key Laboratory for Agricultural Pest Management of Mountainous Region, Institute of Entomology, Guizhou University. *Tetranychus urticae* was reared on kidney bean plants, *Phaseolus vulgaris* L. *Euseius nicholsi* mites were reared with *T. urticae* on fresh kidney bean leaves on rearing platforms in Petri dishes (9 cm diameter). The rearing platform was a circular moist sponge (9 cm diameter) covered with a black cloth (8 cm diameter) and then a plastic wrap (7 cm diameter) on the cloth. The dish with the platform was approximately half-filled with water to prevent the predator from escaping. The lid was removed to allow for ventilation. The Petri dishes were kept at 25 ± 1 °C, 60–70% RH and L16:D8 photoperiod in a climate controlled chamber. A few cotton fibers are placed on the platform surface for female predator oviposition. To allow the emerged larvae to develop in synchrony, newly laid eggs were collected after 6 h and transferred to a fresh dish using a fine paintbrush. The newly emerged larvae and adults were used in the experiments.

### Fluorescein diacetate (FDA)

The liquid fluorescent dye for the experiments described below was prepared by dissolving 4 mg of fluorescein diacetate (Sigma Chemical) in 1 mL of acetone. FDA was stored in an opaque flask at 4 °C. FDA liquid can retain its reactivity for up to 6 months when stored under these conditions (Jones and Sneft 1985). The working solution was prepared by adding 35 μl of FDA liquid to 4 ml of deionized water. Fresh solutions were prepared for each experiment. To protect the solution from light during usage, the containers were wrapped with aluminum foil and placed on ice over an opaque background.

### Infectivity of aerial and submerged conidia to *Tetranychus urticae* and *Euseius nicholsi*

Infectivity of the aerial and submerged conidia of *I. cateniannulata* to *T. urticae* and *E. nicholsi* was re-evaluated with the method by Zhang et al. (2014). The newly emerged female adults of *T. urticae* were inoculated by immersion for 5 s in 2 ml suspension of submerged or aerial conidia, and then carefully transferred using a fine paintbrush onto an excised fresh bean leaf. The bean leaf has previously been placed on top of a water-saturated filter paper in a 9-cm Petri dish, with the root vein of the leaf wrapped with moistened cotton wool to retard desiccation. Dishes were then sealed with their lids, placed into a plastic box (1 l) and incubated at 25 ± 1 °C, 100% RH and L14:D10 photoperiod in a climate controlled chamber. The status of all individuals was determined for 7 days after treatment, with infection progress recorded daily. The randomized treatments consisted of 4 replicates with 30 mites per replicate. The control mites (CK) were treated with sterile water and 0.05% Tween-80 in sterile water, respectively.

The treatments of *E. nicholsi* were identical to the above, except sufficient *T. urticae* larvae were added to each dish as prey. All dead *T. urticae* and *E. nicholsi* were removed and cultured at 25 ± 1 °C, 80% RH under continuous darkness, and then examined for the development of mycelia of *I. cateniannulata* under an optical microscope (Nikon, ECLIPSE *Ti*-*U*).

### Effect of aerial and submerged conidia on the longevity and oviposition of *Euseius nicholsi*

The experimental units were designed using Petri dishes (9 cm diameter) and conditions were the same as those used for the predator colony. Each dish served as a separate experimental platform. The newly molted female adults were inoculated by immersion for 5 s in 2 ml of spore suspension. Each mite was reared in a separate dish with 10 *T. urticae* larvae supplied as food every day. After a male was added to each dish and allowed to mate for 1 day, the male was removed. The oviposition period, the daily fecundity of the successfully mated female mite and the number of eggs hatched were recorded until all females died. The female predator mites and remaining spider mites were transferred into a new dish daily, preventing the female predator from feeding on her own eggs. Aerial and submerged conidia controls were treated with sterile water and sterile water containing 0.05% Tween-80, respectively. In total 280 synchronized female predators were tested, using randomized treatments. The experiment consisted of 3 replicates with 40 mites per replicate and 40 for control. In total, there were 120 predator mites infected by aerial conidia and 120 by submerged conidia.

### Infection process observations made by scanning electron microscopy (SEM) and fluorescence microscopy (FM)

For the SEM observations, specimens of each mite species were inoculated by immersion for 5 s in 2 ml of each of the two spore suspensions, and then separately reared by the method described above. After 1, 2, 6, 12, 24, 36, 48, 60 and 72 h, the treated samples were mounted on SEM stubs using double-sided carbon tape. Dried samples were sputter coated with carbon (HUS-5GB) and gold (JFC-1600) using a high vacuum evaporator and then observed with the SEM (JSM-6490LV).

For the FM observations, specimens of each mite species were inoculated and reared as above. After 1, 2, 12, 24, 36, 48, 60 and 72 h, the treated samples were stained with fluorescein diacetate (FDA) and observed under blue light at a wavelength of 450–490 nm with an inverted microscope (Nikon, ECLIPSE *Ti*-*U*). Photos were taken using a digital camera controller (Nikon Digital Sight PS-U3).

### Statistical analysis

All statistical analyses were carried out using SPSS v.17.0. Significance of mortality differences between the two species was evaluated using a *t* test at a significance level of 0.05. Differences in the longevity and oviposition values between the treatments and controls of *E. nicholsi* were also compared by *t* test after log transformation of the data. Corrected mortality rate (%)  = (number of test mites − average survival of treatment + average number of deaths in control)/number of total mites tested] × 100%.

## Results

### Effect of aerial conidia and submerged conidia on *Tetranychus urticae* and *Euseius nicholsi*

**Fig. 1:**
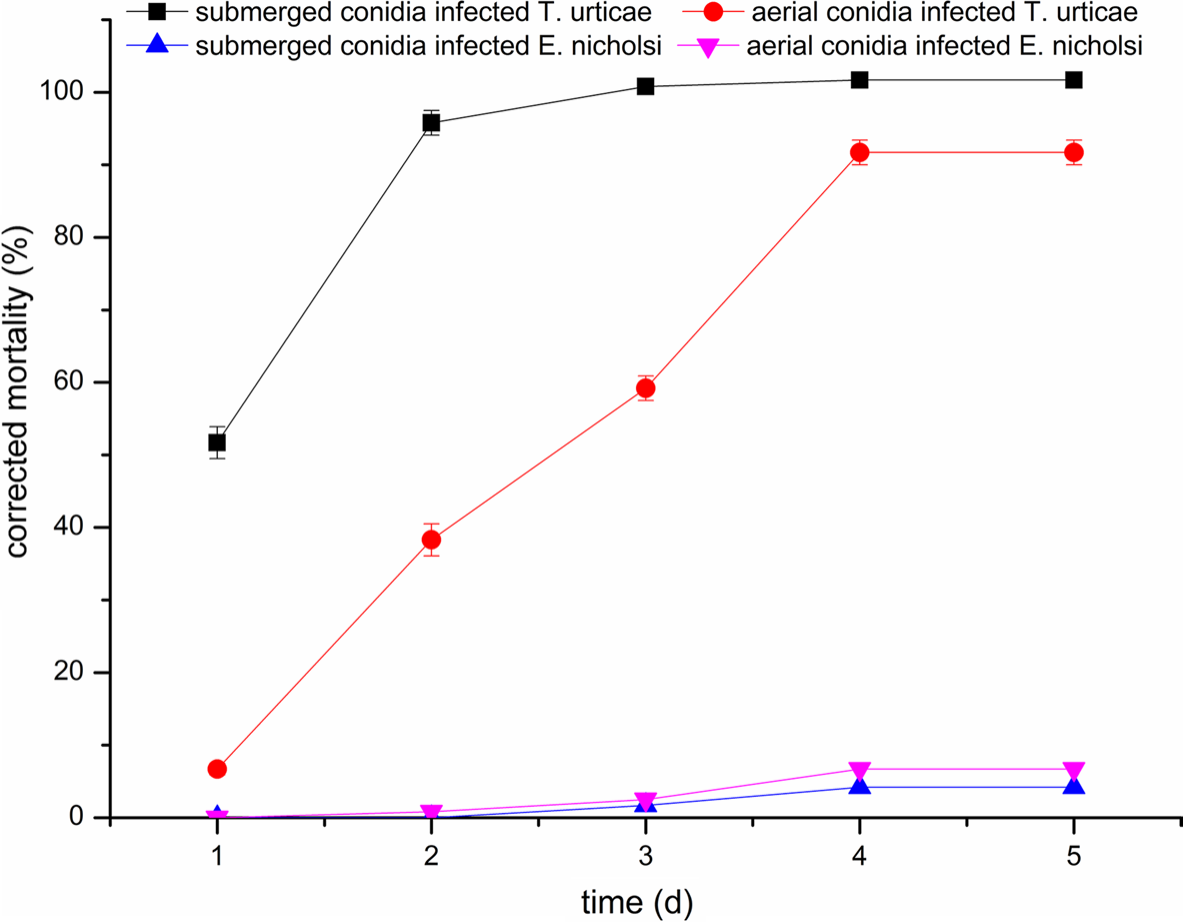
Mortality (%) of *Tetranychus urticae* and *Euseius nicholsi* infected by aerial and submerged conidia of *Isaria cateniannulata* over time (days)

The mortalities of *T. urticae* and *E. nicholsi* infected by aerial and submerged conidia, increased with time during the first 3 days and then remained constant (Fig. 1). The mortality of *T. urticae* infected by submerged conidia reached 100% at day 3, and was higher than when treated with aerial conidia, peaking at 92% on day 4. The highest mortalities of *E. nicholsi* infected by submerged and aerial conidia were as low as 4.2 and 6.7%, respectively, occurred on the 4th day.

Table 1 demonstrates that there were no significant differences between the treatments and the controls with either aerial or submerged conidia of *I. cateniannulata* on the lengths of the preoviposition, oviposition, or postoviposition periods, and the female longevity or the daily fecundity of *E. nicholsi*. It is suggested that *I. cateniannulata* has no definitive effect on the vitality and fertility of *E. nicholsi*.

**Table 1:** Effect of aerial and submerged conidia on mean (± SE) oviposition, longevity and fecundity of *Euseius nicholsi*

Treatments and index	Conidia suspension	Preoviposition (days)	Oviposition (days)	Postoviposition (days)	Female longevity (days)	Daily fecundity (no. eggs/mite)
Controls		1.537 ± 0.054a	18.604 ± 0.074a	7.460 ± 0.055a	26.633 ± 0.473a	1.543 ± 0.064a
Treatments	Submerged	1.551 ± 0.029a	18.562 ± 0.038a	7.409 ± 0.032a	26.578 ± 0.302a	1.497 ± 0.027a
	Aerial	1.517 ± 0.028a	18.638 ± 0.031a	7.464 ± 0.030a	26.233 ± 0.282a	1.449 ± 0.272a
*t* (d.f. = 89)	Submerged	0.530	− 1.087	− 1.592	− 0.183	− 1.715
	Aerial	− 0.685	1.079	0.150	− 1.416	− 1.138
*P*	Submerged	0.60	0.28	0.12	0.86	0.090
	Aerial	0.50	0.28	0.88	0.16	0.26

Means within a column followed by the same letter are not significantly different (*t* test: *P *< 0.05)

### The distribution of *Isaria cateniannulata* spores on *Tetranychus urticae* and *Euseius nicholsi*

**Fig. 2:**
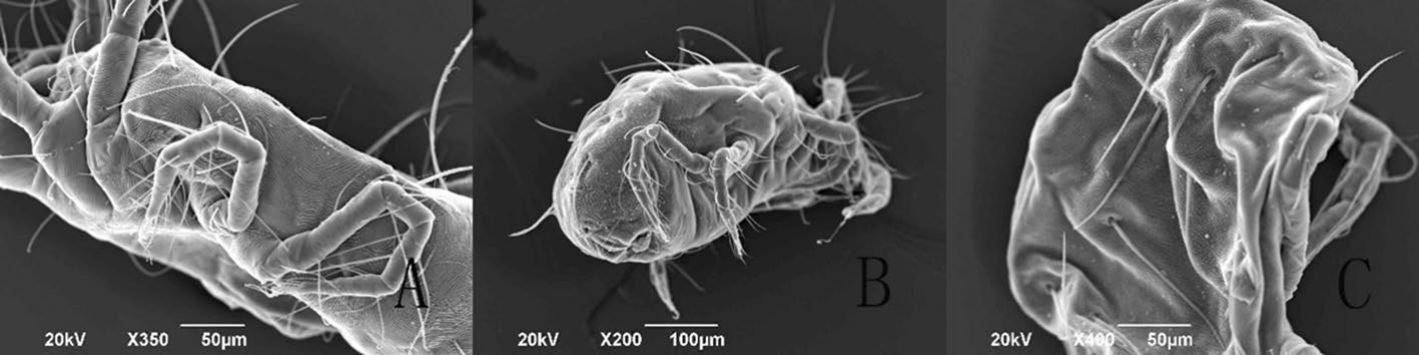
Conidia of *Isaria cateniannulata* distributed on *Tetranychus urticae* body in a different view

**Fig. 3:**
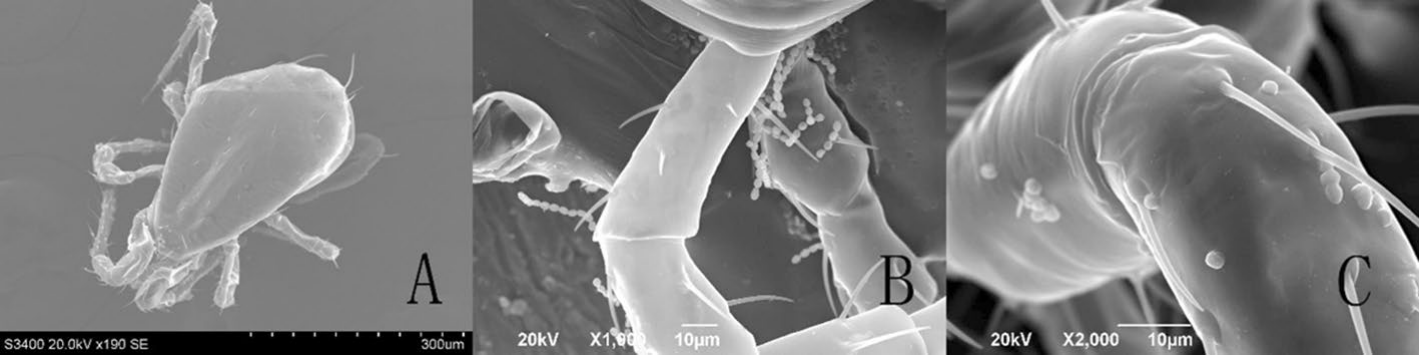
Distribution of *Isaria cateniannulata* spores on *Euseius nicholsi*. **a** Dorsal view; **b**–**c** leg I

**Fig. 4:**
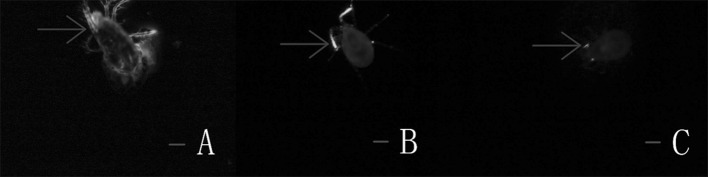
Distribution of *Isaria cateniannulata* spores, the brightly colored areas, on *Tetranychus urticae* and *Euseius nicholsi*. *Scale bar* 100 µm. **a** *T. urticae*; **b** *E. nicholsi*; **c** leg I of *E. nicholsi*

Observed at 1 h after treatment, the spores, aerial conidia and submerged conidia, were evenly distributed on the entire body of *T. urticae* under the SEM (Fig. 2) and FM (Fig. 4a). In *E. nicholsi*, however, the spores were restricted mainly to legs I (Figs. 3b, c, 4b, c).

### Infection behavior of *Isaria cateniannulata* under scanning electron microscopy

#### Infection/growth behavior of submerged conidia on *Tetranychus urticae*

**Fig. 5:**
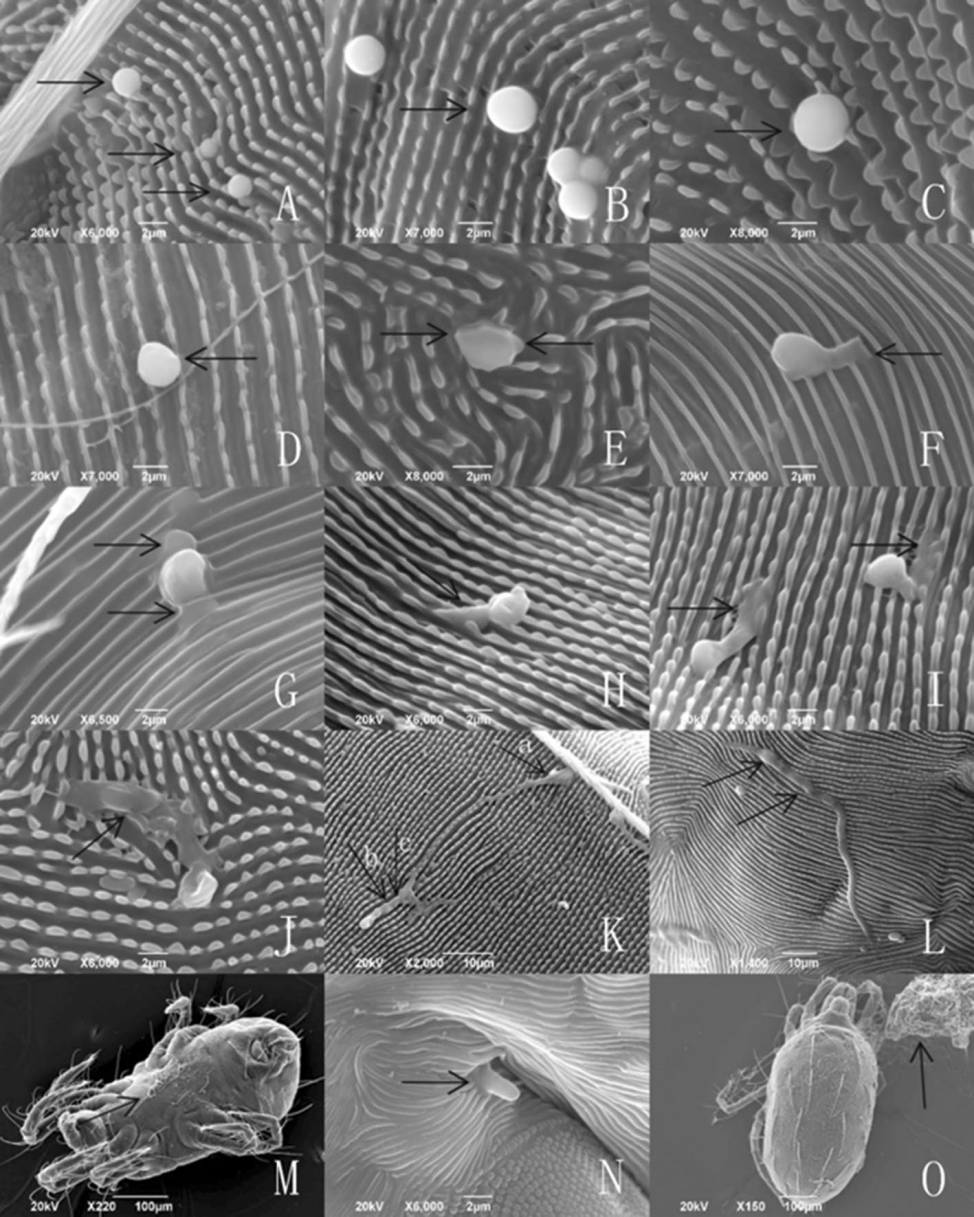
Infection sequence of *Isaria cateniannulata* submerged conidia on the cuticle of *Tetranychus urticae.* **a** Submerged conidia (diameter ≤ 2 μm) attached to cuticle 1 h after treatment. **b** Submerged conidia beginning to swell, at ≥ 1 h. **c** Submerged conidia are significantly swollen (diameter > 2 μm), at ca. 2 h. **d** Some submerged conidia become deflated and their germ tubes appear stunted, at 4 h. **e** Appearance of the germ tubes from different views, at 4 h. **f** A germ tube from a submerged conidium developing toward the cuticle. **g** Two germ tubes from a single conidium developed towards the cuticle. **h** A submerged conidium directly developing a penetration peg to penetrate the cuticle, at 24 h. **i** A germ tube repositioning itself to ‘find’ a ‘proper’ site for infecting, at 24 h. **j** A germ tube oriented in place and transferred its nucleus into the mite. **k** A germ tube (**a**) repositioning itself and combined with another conidium to penetrate the cuticle (**b**, **c**), at 24 h. **l** Two germ tubes of a conidium spreading in two directions. **m** General growth state of the fungus on the mite venter after 24 h. **n** Mycelium growth from the mite body after 48 h. **o** Mycelium colonizing the entire mite body after 72 h

The conidia adhering to the surface of female *T. urticae* were unaltered after 1 h (Figs. 2 and 5a), but began to swell as time increased to 2 h (Fig. 5b). At 4 h, some conidia become deflated and their germ tube began to faintly appear (Fig. 5c). After 12 h, the germ tube was highly developed and at the correct position to infect. Some conidia had developed a penetration peg to infect at 12 h, at which time mite movement was noticeably slowed. The mite ceased walking at approximately 24 h. Mycelia emerged from the mite body after 48 h and covered the entire body by 72 h.

**Fig. 6:**
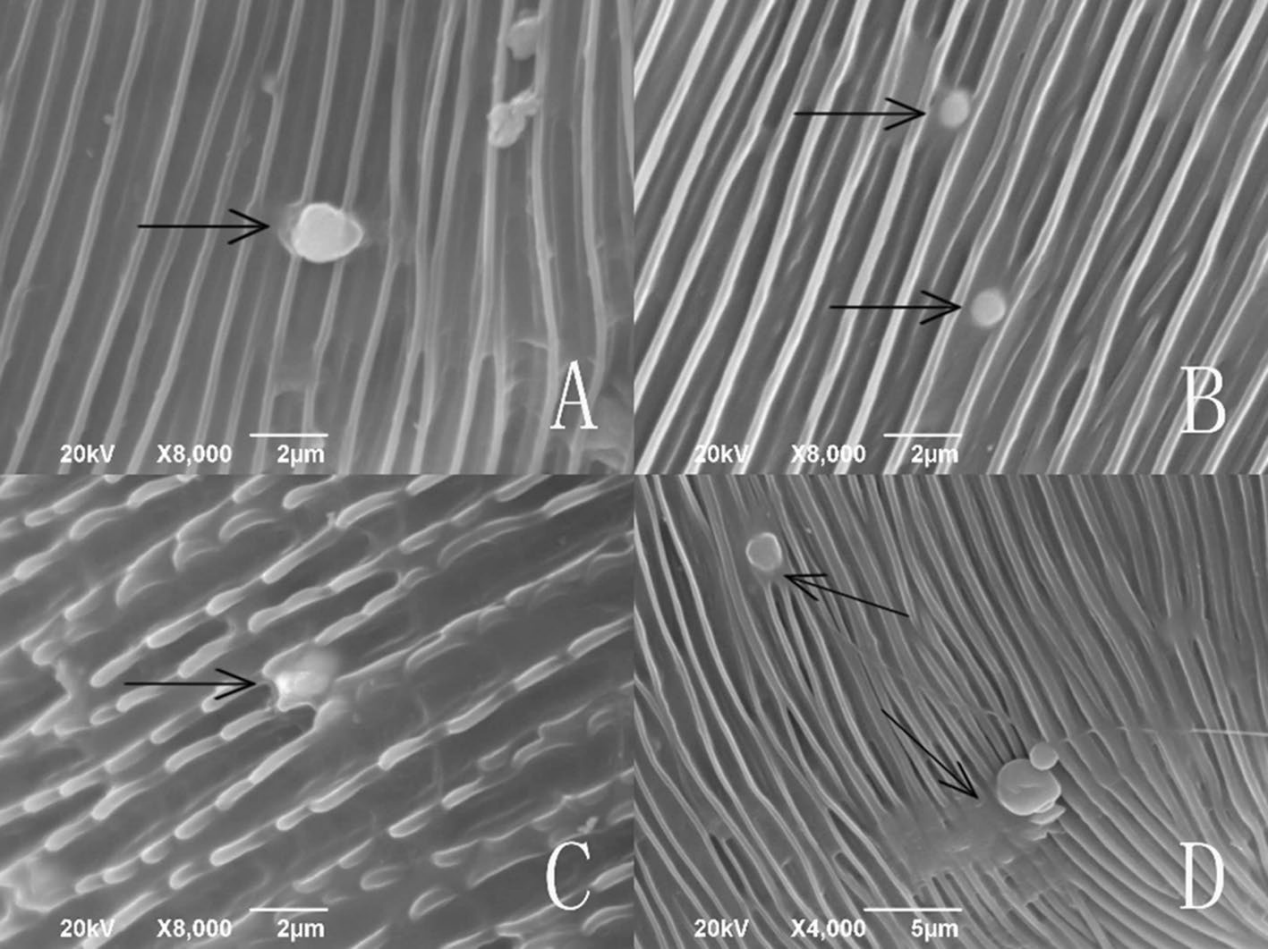
Submerged conidia and secretion on the cuticle of *Tetranychus urticae*

**Fig. 7:**
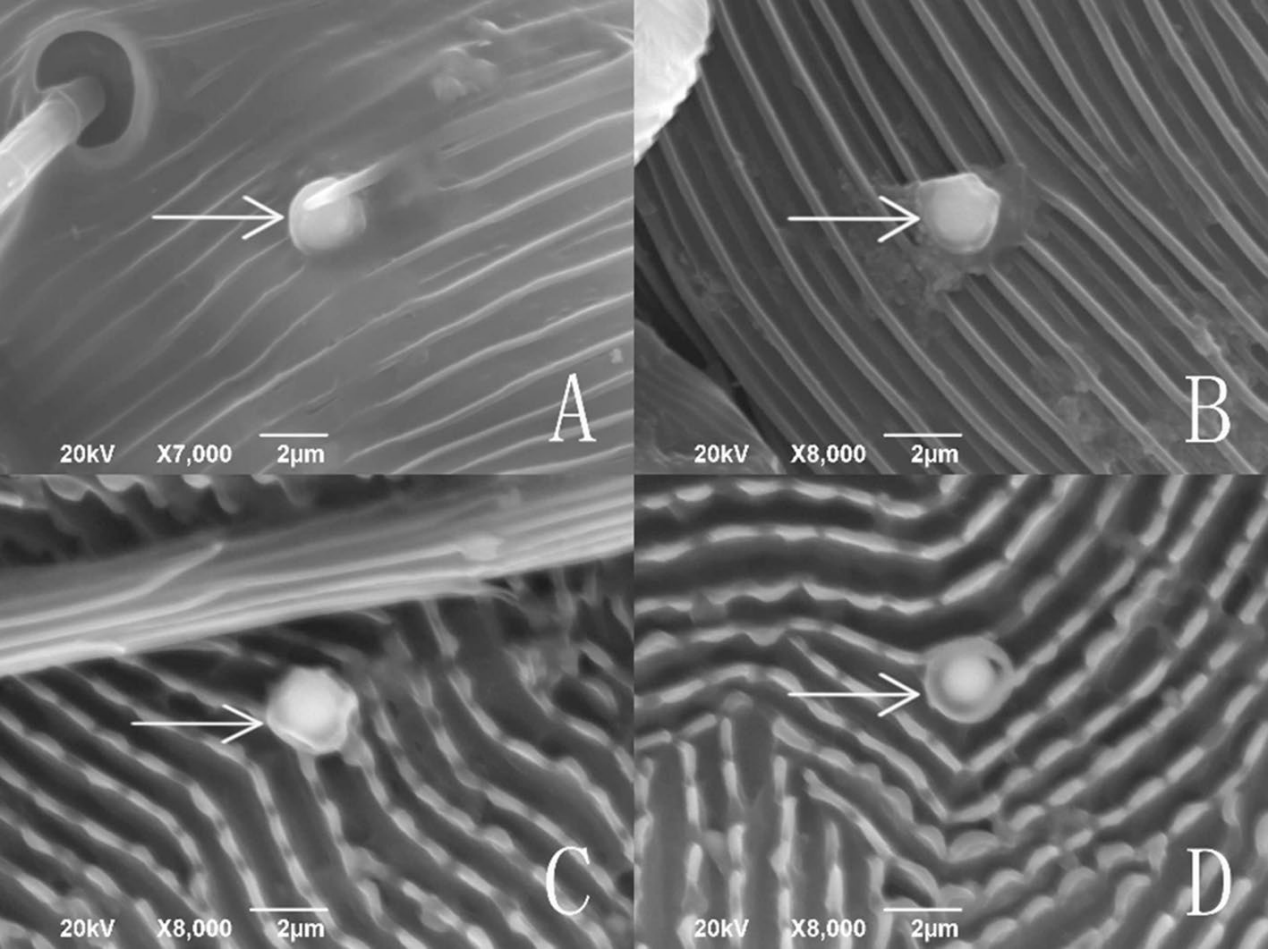
A submerged conidium with its pellicle separated from sclerotium on the cuticle of *Tetranychus urticae*

Some submerged conidia were unique in releasing a substance on the interface between the conidium and the cuticle after 2, 4, 8, and 16 h of the treatment (Fig. 6a–d, separately). These conidia, however, did not develop further. It was also observed that the pellicle separated from the sclerotium in some submerged conidia after 2, 4, 16 and 20 h (Fig. 7a–d, separately).

#### Infection/growth behavior of the aerial conidia on *Tetranychus urticae*

**Fig. 8:**
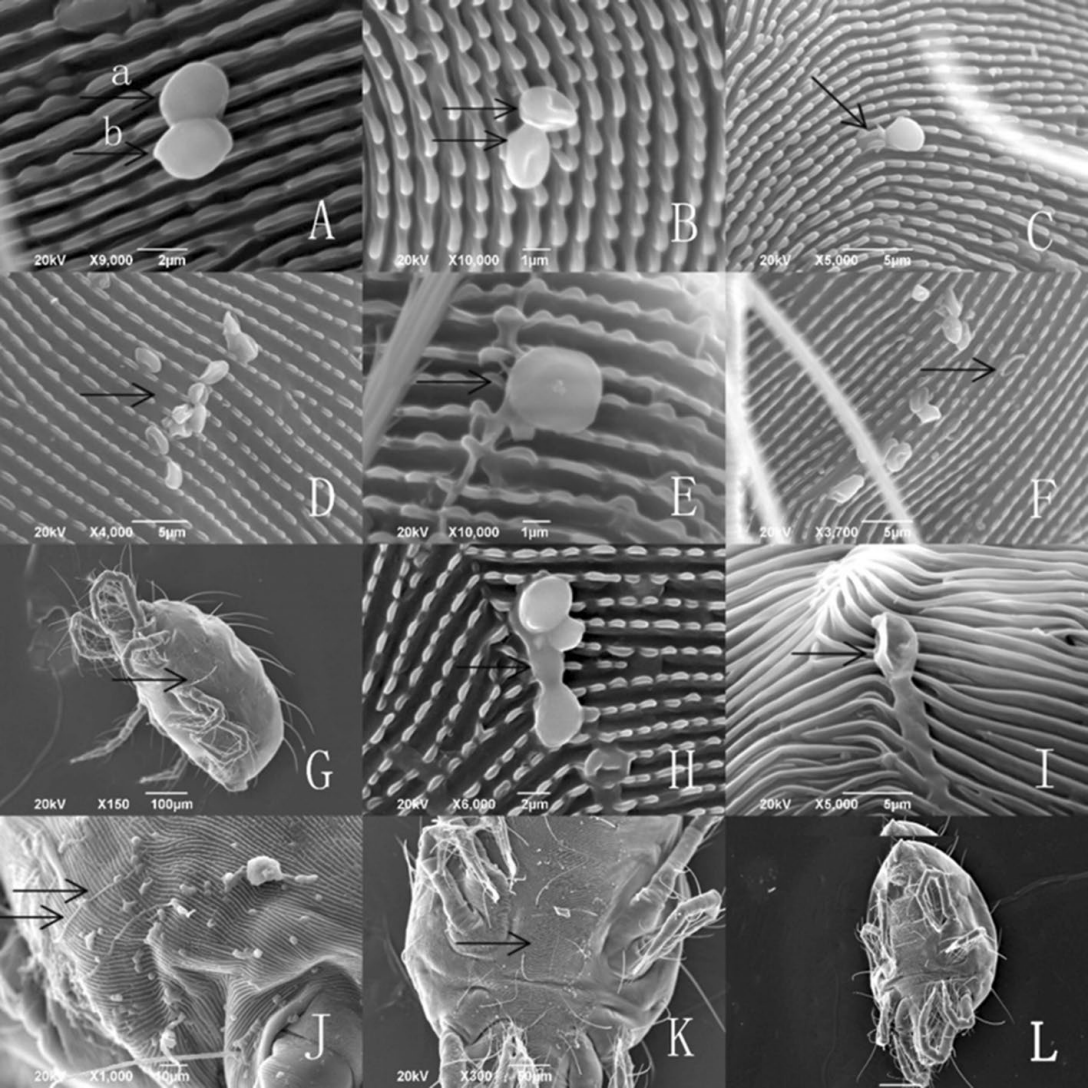
Infection sequence of *Isaria cateniannulata* aerial conidia on the cuticle of *Tetranychus urticae.* **a** Aerial conidia (diameter ≥ 2.5 μm) adhering to *T. urticae* cuticle at 1 h after treatment. **b** Aerial conidia beginning to shrivel after 4 h. **c** Germ tube of an aerial conidium appeared on one side in 4 h. **d** Most of aerial conidia shriveled after 16 h. **e** Shriveled aerial conidia without sclerotia. **f** Shriveled aerial conidia, at 24 h. **g** Live mite with shriveled aerial conidia. **h** Germ tube of an aerial conidium developed toward the cuticle and correctly oriented for infecting, at 36 h. **i** Nucleus transferred into the mite, at 48 h. **j** An area with numerous shriveled conidia and several developed conidia, at 72 h. **k** Mycelium colonizing the mite body, at 96 h. **l** Control cadaver of *T. urticae* treated with sterile water or sterile water with 0.05% Tween-80

At 1 h, aerial conidia were observed adhering to the surface of female *T. urticae*. Afterwards, most of the conidia shriveled, and only a few were developed at 4 h. Most conidia had no sclerotium and could not develop further with time. After 36 h, a few conidia developed a germ tube to search for an appropriate place to infect, and subsequently developed the penetration peg to infect at 48 h. The infected mites began moving slowly by that time, and then ceased walking entirely at approximately 72 h. The mycelium covered the entire mite body at 96 h (Fig. 8a–l).

#### Infection/growth behavior of submerged and aerial conidia on *Euseius nicholsi*

**Fig. 9:**
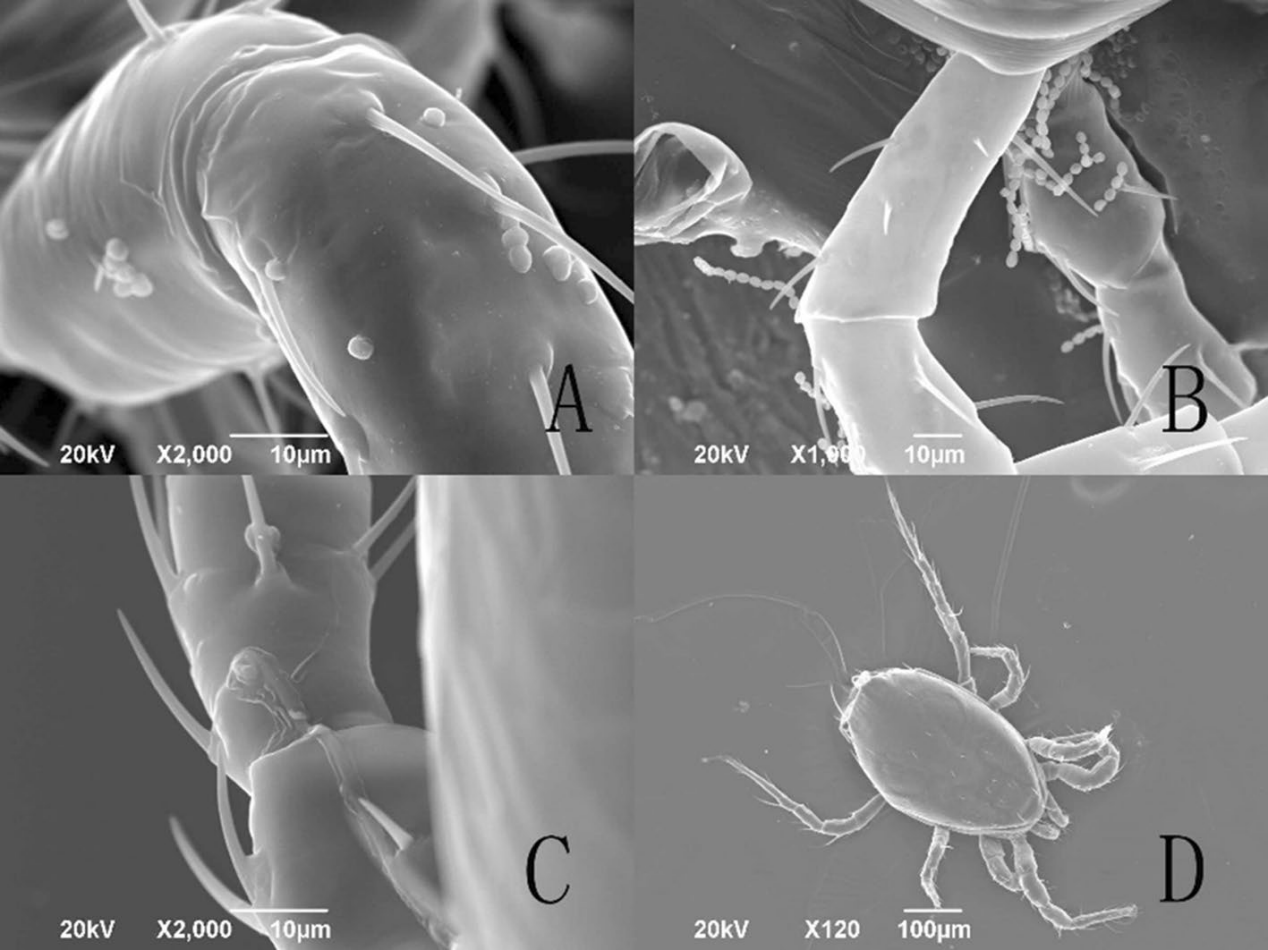
Infection sequence of *Isaria cateniannulata* aerial and submerged conidia on the cuticle of *Euseius nicholsi*. **a** Submerged conidia adhering to the mite cuticle, at 1 h. **b** Submerged conidia adhering to the mite, at 1 h. **c** Conidia development on the mite. **d** Control cadaver of *E. nicholsi* treated with sterile H_2_O or sterile water with 0.05% Tween-80

At 1 h after treatment with aerial and submerged conidia, the spores were only found adhering to legs I of *E. nicholsi*. A few conidia succeeded in developing further but the infected mites rarely died (Fig. 9a–d).

#### Fluorescence microscope observation of infection behavior of *Isaria cateniannulata*

**Fig. 10:**
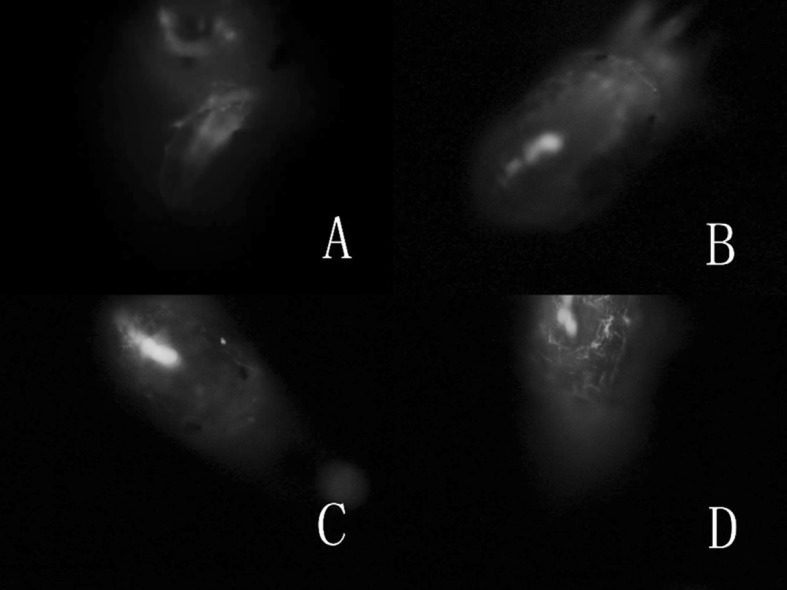
The FM showed infection status of *Isaria cateniannulata* aerial and submerged conidia in *Tetranychus urticae*. **a** Penetration of the germ tube and hyphal growth within the mite body (8 h with submerged conidia, 12 h with aerial conidia). **b, c** Hyphae propagating. **d** Spore differentiation (20 h with submerged conidia and 40 h with aerial conidia)

It was observed under FM that the germ tubes penetrated the cuticle and hyphae grow from the body of *T. urticae* females infected by submerged conidia and aerial conidia (Fig. 10, 6–24 h).

## Discussion

Although the entomopathogenic fungus *I. cateniannulata* is capable of producing submerged conidia in liquid medium and aerial conidia on solid medium (Zhang et al. 2014), determining which type is best for commercial formulations may be difficult. For example, a study comparing the bioactivity of *B. bassiana* aerial and submerged conidia has shown that they have similar virulence toward insects (Bidochka et al. 1995; So 2005). Bugeme et al. (2015) and Jenkins and Goettel (1997) showed that the diphasic liquid–solid fermentation system appeared to be promising in the mass production of mycoinsecticides based on aerial conidia of *Metarhizium* for insect control. Considering that production of aerial conidia, either through surface cultivation or a two-stage process, requires more time, more space and more labor (Hall and Papierok 1982), Fargues et al. (2001) suggested that an appropriate shake-flask culture medium would provide submerged conidia as economically produced mycoinsecticides for commercial development. Submerged conidia could be produced in large volume using inexpensive and readily available substrates (Dwayne et al. 1992). In the present study, we have shown that, based on their virulence to the two-spotted spider mite, submerged conidia clearly produced superior results over aerial conidia in controlling spider mite populations.

From the observations by SEM and FM on the infecting behavior and capability of submerged versus aerial conidia, *I. cateniannulata* could effectively infect *T. urticae* but not the predatory mite *E. nicholsi*. It was evident from the characteristic bright color observed under FM, that infections were widespread and generalized over the entire body of *T. urticae* (Fig. 4), but in *E. nicholsi*, only the anterior legs were covered with the brightly colored spores. In *T. urticae*, the whole infection process was clearly observable (Figs. 5 and 8) and could be generalized as five stages: (1) Conidia settlement; (2) Conidia germination; (3) Germ tube and penetration peg development; (4) Penetration; (5) Mycelia developing within the body and then emerging from the host body. Several atypical growth traits of the fungus were also noted: (1) Some submerged conidia would develop two distinct germ tubes which would then spread in different directions (Fig. 5l); (2) Some submerged conidia produced a secretion at the interface between the conidium and the mite cuticle (Fig. 6); (3) Some submerged conidia had their pellicle separated from the sclerotium (Fig. 7), which might be due to unsuitable micro-conditions; (4) Most aerial conidia shrivelled with only a few germinating on *T. urticae* (Fig. 8); (5) Both the aerial and submerged conidia mostly did not develop on *E. nicholsi*, and a few germinated had no apparent lethality to the predatory mites (Fig. 9).The infected female *T. urticae* had a visible bead-shaped ‘light’ inside body (Fig. 10b, c), which might be the immature eggs infected by *I. cateniannulata*.

In general, the infection process by entomopathogenic fungi can be summarized as follows. The conidia attach to the host cuticle, followed by germinating and producing the infection structure. The hyphae then penetrate the cuticle and proliferate in the host mite body. The host eventually dies (Pu and Li 1996). In each of these stages, recognition and interaction between the pathogen and its host may be involved in successful infections characterized by the degree of virulence of the fungus to the host. Compared to projection electron microscopy with paraffin sectioning for observing the infection progress, the SEM and the FM methods used herein appear to be superior in saving time, labor and material expenses.

Conidia of entomopathogenic fungi generally display a wide range of surface physicochemical properties that allow them to interact and adhere to substrata, and the hydrophobicity of the conidia surface often associated with virulence (Holder et al. 2007; Dwayne et al. 1992). The hydrophobic property of the conidia are determined by their various surface features. For example, *Nomuraea rileyi* (Farlow), *M. anisopliae* and *I. fumosorosea* have been proved to have hydrophilic outer rodlet layers. *Hirsutella thompsonii* Fisher and *Verticillium lecanii* (Zimmerman) are characterized by lack of a rodlet layer, but they have an outer mucilaginous coat produced during spore maturation (Drion et al. 1991). In this study, the submerged conidia produced a secretion on the interface between the submerged conidia and the mite’s cuticle. We theorize that the submerged conidia of *I. cateniannulata* may be hydrophilic, but lack a rodlet layer, whereas the aerial conidia may be hydrophobic. This hypothesis needs to be substantiated in future studies.

In general, the two types of conidia are capable of infecting the two-spotted spider mite but not the predatory mite. Mechanisms involved in this phenomenon may be attributed to the behavior and the cuticle traits. It has been reported that the predatory mites are efficient in removing most of the capilliconidia of the fungal pathogen through self-grooming behavior (Wekesa et al. 2007; Wu et al. 2016a, b), but no comparable behavior has been observed in *T. urticae*. The structure and chemical composition of the cuticle in the two-spotted spider mite differ substantially from that found in the predaceous species (Pu and Li 1996). It has been demonstrated that the cell surface of aerial conidium is hydrophobic and that of submerged conidia is borderline between hydrophobic and hydrophilic, which are concerned with host cuticle traits (Holder et al. 2007), i.e., it may be hydrophobic or hydrophilic depending on the cuticle feature. It is suggested to explore the cuticle traits of *T. urticae* and *E. nicholsi* in future studies.

The potential detrimental side effects resulting from inopportune applications of pathogens and/or predators to control pests have frequently drawn scientists’ attention. Furtado et al. (1996) reported that a strain of the fungal pathogen *Neozygites acaricida* (Petch) was pathogenic to the predaceous phytoseiid mite *Euseius citrifolius* Dernmark & Muma, and Wekesa et al. (2007) observed that *Neozygites floridana* (Weiser & Muma) could reduce egg predation of the phytoseiid mite *Phytoseiulus longipes* Evans. Other studies, however, have demonstrated that some other fungal pathogens had no apparent effect on predatory mites (Jacobson et al. 2001; Wang et al. 2011; Wu et al. 2014; Ullah and Lim 2017). The present study, as well as our previous research (Zhang et al. 2016), has confirmed that *I. cateniannulata* has no deleterious effects on the vitality and fertility of the predator *E. nicholsi*.

In conclusion, SEM and FM observations proved that submerged conidia are clearly more effective than the aerial conidia in infecting *T. urticae*. Neither submerged nor aerial conidia had any significant effect on the preoviposition, oviposition, postoviposition, daily fecundity or longevity of females of *E. nicholsi*, suggesting that both types of propagules are safe to use for *E. nicholsi.* These results indicate that the two biological control agents, *I. cateniannulata* and *E. nicholsi*, may be compatible candidates applied concomitantly in the biocontrol programs of *T. urticae*.
